# Incidence of Health and Behavior Problems in Service Dog Candidates Neutered at Various Ages

**DOI:** 10.3389/fvets.2019.00334

**Published:** 2019-10-08

**Authors:** Marta Zlotnick, Virginia Corrigan, Erin Griffin, Megan Alayon, Laura Hungerford

**Affiliations:** ^1^Population Health Sciences, Virginia Maryland College of Veterinary Medicine, Virginia Tech, Blacksburg, VA, United States; ^2^Center for Animal Human Relationships, Virginia Maryland College of Veterinary Medicine, Virginia Tech, Blacksburg, VA, United States; ^3^Virginia Maryland College of Veterinary Medicine, Virginia Tech, Blacksburg, VA, United States

**Keywords:** gonadectomy, spay, neuter, service dogs, dogs, orthopedics, behavior

## Abstract

Saint Francis Service Dogs (SFSD) trains dogs to aid people with multiple sclerosis, brain injury, and many other conditions. Organizations like SFSD must carefully consider when to neuter dogs to give them the best chance at successfully completing lengthy and expensive training. The objective of this retrospective cohort study was to assess differences in the incidence of health or behavior problems leading to dismissal between dogs neutered at different ages. Data on 245 dogs—including birth date, sex, neuter date, dismissal or successful completion of training, and (where applicable) reason for dismissal—were collected from SFSD records. Age-at-neuter was grouped (<7 months; 7–11 months; > 11 months) and compared for dogs who successfully completed training and dogs who were dismissed. Dogs neutered from 7 to 11 months of age were dismissed at a significantly lower overall rate than dogs neutered at an older or younger age. There were no differences between males and females. Labrador and golden retrievers were less likely to be dismissed than other breeds. This pattern was the same for dismissals for behavioral reasons. Dogs neutered at <7 months had more than twice the risk for health-related dismissals as dogs neutered at any older age and this pattern held for orthopedic dismissals. Labradors were at higher risk for orthopedic-related dismissal than golden retrievers and all other breeds. This study suggests that there is a relationship between dogs' age at neuter and the incidence of health and behavioral problems that can lead to dismissal from service dog training.

## Introduction

In general, pet owners are encouraged to spay or neuter their dogs in order to reduce the incidence of accidental litters and improve some aspects of pet health. Reported risks and benefits of gonadectomy may include effects on musculoskeletal problems, neoplasia, behavior, urinary incontinence, weight, or lifespan (1–7). However, questions remain about the safety and long-term effects of the gonadectomy procedure (spay/neuter) for animals at a young age, despite considerable literature on the subject, some of which is contradictory. In addition to many pet dogs in the U.S., dogs who act as service animals are often gonadectomized; Assistance Dogs International, an accreditation organization, requires that service dogs be spayed or neutered in its training standards (8).

Examinations of the effects of gonadectomy in service dog training programs are rare. Unlike pet dogs, service dogs are judged on their ability to successfully complete training requirements, in addition to meeting standards of health and behavior. Kustritz has stated that age at gonadectomy did not alter trainability of working dogs (9), but more detailed examinations of service dogs-in-training are critical to our understanding of the needs of future service animals.

Saint Francis Service Dogs (SFSD) is a non-profit organization in Roanoke, Virginia, USA, which trains service dogs for children and adults with disabilities. Founded in 1996, SFSD has placed dogs with people with disabilities and in locations such as courtrooms and healthcare facilities since 1998. Puppies of about 8 weeks of age, typically donated by private breeders, are placed with a puppy-raiser—usually in a private home—and taught basic manners and commands until ~15 months of age, at which time they are transferred to a trainer to begin 9 to 12 months of formal training as a service dog.

Until 2013, most puppies in the SFSD program were spayed/neutered at ~4 to 6 months of age. Gonadectomy at this early age has commonly been recommended to prevent accidental litters and reduce the risks of cancers of the reproductive organs, and in large-breed dogs, puberty has not yet occurred at this time. For example, golden retrievers typically experience puberty between 9 and 11 months of age (10). However, concerns have developed about the long-term effects of early-age neutering, including increased risks of cystitis, hip dysplasia, and urinary incontinence (11). Beginning in ~2014, due to a change in SFSD policy at the suggestion of breeders, dogs in the program were spayed/neutered at ~1 year of age, after most of the dogs had reached puberty. This situation provided an opportunity in a group of similarly-managed animals to assess differences in the incidence of health or behavior problems between dogs neutered at different ages, as well as the animals' ability to complete service dog training.

## Materials and Methods

Information from the files of all dogs who participated in the SFSD training program between 1996 and 2017 were examined for this study. Whelp date, date of neuter (including castrations and spays), sex, breed, the cause of dismissal for each of the dogs, and details about the dog's dismissal—such as a behavior trigger or medical test performed—were abstracted into a spreadsheet [Excel]. Dogs were excluded from the study if less than 3 months had elapsed between their date of neuter and their dismissal or graduation from the training program, or if dogs were not neutered until after either completion of training or dismissal. This was an effort to eliminate any dogs who might have already had signs of hip dysplasia at the time of neuter. Dogs were also excluded if records were missing crucial variables needed for analysis.

Two general categories for dismissal were established: behavior problems (including behaviors that caused training difficulties) and health problems. Health reasons for dismissal were diagnosed by a veterinarian. Determinations of behavioral and training-related difficulties were made by a trainer. Some dogs were dismissed from the SFSD training program for reasons that included both a health and a behavior problem. This group was reported separately in descriptive analyses but included in each dismissal group when compared separately to dogs that graduated. For the purposes of this study, the terms “graduation” or “successful” dog referred to a dog that completed the SFSD training program.

Labrador retrievers and golden retrievers included in the analysis were considered their own groups. All other breeds and mixed-breed dogs were grouped into an “Other” breed group due to limited numbers.

Dogs were divided into three groups to compare the age-at-neuter periods most often used by SFSD (<7 months and >11 months), as well as the time period that covered the ages between the target neuter ages (7–11 months). These age-at-neuter groups also align with some current recommendations for early neutering (12) and with physiological milestones, such as the onset of puberty and closure of growth plates in the long bones (13) at about 12 months of age. Delays in closure may result in increased long bone length and possible alterations to a dog's normal conformation.

The proportion of dogs that graduated or were dismissed for behavioral, orthopedic, or other health reasons was determined for the three age-at-neuter groups and also for other variables (sex and breed) that were potential confounders. To further characterize the relationship of age-at-neuter with each outcome, the median and inter-quartile range were also determined (because age-at-neuter was not normally distributed).

Confounding and effect modification were first assessed by stratifying the analysis of age-at-neuter and dismissal by sex and breed [(14); SAS 9.4®, PROC FREQ] and presented graphically. Multivariable log-binomial regression was used to estimate the risk for dismissal by age-at-neuter group, while examining potential for confounding or effect modification by sex and breed. This type of regression was used because the dichotomous outcome of interest was not rare [(15); SAS 9.4®, PROC GENMOD]. Statistical significance was set at *p* ≤ 0.05. Because this was a retrospective study, and no dogs were handled or manipulated in the execution of this study, no IACUC approval was required.

## Results

Of the 357 dogs who had training records during this period, 21 dogs were excluded because they were neutered less than 3 months before dismissal. An additional 91 dogs were excluded from analysis because their records lacked sufficient information on key variables, most commonly about the cause of their dismissal, whelp date, and/or date of neuter. Of the 245 dogs in the study, 110 (45%) were neutered at less than 7 months of age, 58 (24%) between 7 and 11 months of age, and 77 (31%) when older than 11 months of age. The median age-at-neuter was 244 days (8.1 months) of age (range: 107 to 1,319 days). Two dogs neutered after 3 years of age joined the SFSD program as adults and were in training during and after their gonadectomy. Most dogs were male, and Labrador and golden retrievers were the predominant breed types, although there were other breeds, including purebred dogs (6 Australian Shepherds, 3 Standard Poodles, 2 Smooth Coated Collies, 1 German Shorthair Pointer, 1 Belgium Traverne, 1 Border Collie, and 1 Portuguese Water Dog) as well as 24 mixed-breed dogs (Table 1). Less than half of dogs successfully completed the training program and there were no significant differences in graduation rates between males and females or between breeds (Table 1). Most dismissals were for behavior-related reasons (Table 1, 75% of all dismissals), while 20.5% of all dismissals were for health-related reasons and 4.1% of all dismissals were for simultaneous health and behavior problems.

**Table 1:** Characteristics of dogs that graduated or were dismissed from training from 1996 through 2017 among a cohort of SFSD dogs.

**Demographic characteristics^a^**	**Graduated (*n* = 99)**	**Dismissed for behavior (*n* = 110)^#^**	**Dismissed for orthopedic problems (*n* = 25)^#^**	**Dismissed for other health problems (*n* = 11)^#^**	**Dismissed for behavior and health problems (*n* = 6)**
**SEX**					
Female (*n* = 77)	29 (37.7%)	40 (51.9%)	8 (10.4%)	0 (0%)	3 (3.9%) [3 O, 0 H]
Male (*n* = 168)	70 (41.7%)	70 (41.7%)	17 (10.1%)	11 (6.5%)	3 (1.8%) [2 O, 1 H]
**BREED**					
Labrador Retriever (*n* = 149)	63 (42.3%)	58 (38.9%)	22 (14.8%)	6 (4.0%)	5 (3.4%) [4 O, 1 H]
Golden Retriever (*n* = 57)	25 (43.9%)	27 (47.4%)	3 (5.3%)	2 (3.5%)	1 (1.8%) [1 O, 0 H]
Mixed or Other Breed (*n* = 39)	11 (28.2%)	25 (64.1%)	0 (0%)	3 (7.7%)	0 (0%)
**AGE-AT-NEUTER**					
<7 months of age (*n* = 110)	35 (31.8%)	52 (47.2%)	15 (13.6%)	8 (7.3%)	4 (3.6%) [3 O, 1 H]
7-11 months of age (*n* = 58)	34 (58.6%)	20 (34.5%)	4 (6.9%)	0 (0%)	0 (0%)
> 11 months of age (*n* = 77)	30 (39.0%)	38 (49.4%)	6 (7.8%)	3 (3.9%)	2 (2.6%) [2 O, 0 H]
Continuous (days)	284 (115–1,070)	238 (109–644)	254 (101–1,319)	194 (115–478)	236 (120–459)

a *Actual numbers and row percentages are reported for categorical data. Continuous variables are indicated by reporting median and range for non-normally distributed variables*.

# *Dogs that had both behavioral and orthopedic (O) or other health (H) reasons for dismissal are included in totals for these columns*.

Behavior problems resulting in dismissal included aggression, dominance, snapping/biting, growling, resource guarding, being confrontational, being possessive, hyperactivity, reactive or alarm barking, high prey drive, fear, separation anxiety, weak human bond, or sensitivity to sound. This category also included behavioral problems related to barriers to training or reliable work as a service dog, such as poor focus, distractibility, unpredictability, inconsistency, lack of motivation, slowing down when trainer was in a hurry, poor retrieve drive, poor recall, hesitation or refusal to participate in new or familiar environments, and inability or unwillingness to perform required skills.

Orthopedic health problems that resulted in dismissal (17% of all dismissals) included abnormalities associated with the musculoskeletal system, such as hip dysplasia or poor score on PennHIP (16), elbow dysplasia, osteochondritis dissecans (OCD), cruciate ligament disease, degenerative joint disease (DJD), and/or related structural and functional problems. Non-orthopedic health problems that led to dismissal (7.5% of all dismissals) included cardiac disease, heart murmur, overbite, underbite, seizures, unexplained trembling or weakness, histiocytosis, inflammatory bowel disease (IBD), persistent urinary tract infection, allergies, compromised vision, and/or orthopedic problems.

**Figure 1:**
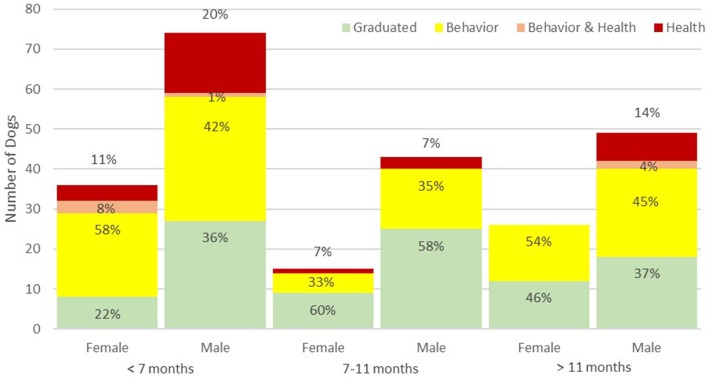
Percent of each training outcome for females and males by age at neutering. Health dismissals are red, behavior dismissals are yellow, simultaneous health and behavior dismissals are orange, and graduations are in green.

**Figure 2:**
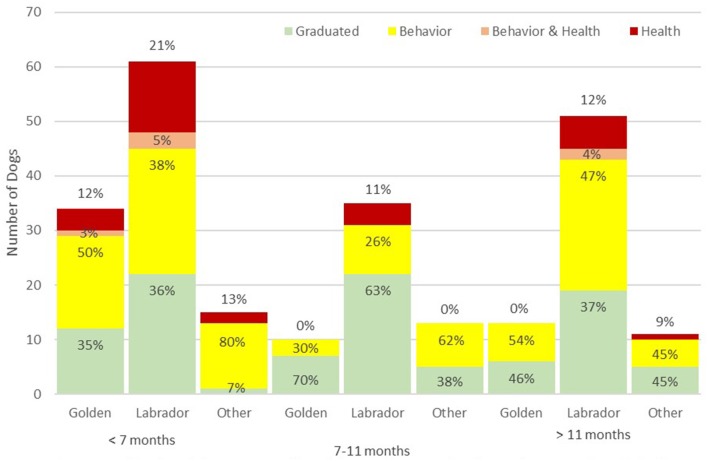
Percent of each training outcome for golden retrievers, Labrador retrievers, and pooled other breeds by age at neutering. Health dismissals are red, behavior dismissals are yellow, simultaneous health and behavior dismissals are orange, and graduations are in green.

Total dismissals and dismissal for behavioral problems followed similar patterns. Neither differed by sex or breed but both were significantly less common among dogs neutered between 7 and 11 months than among dogs neutered before 7 months or after 11 months (Table 1, Figure 1). This pattern of a lower proportion of dogs dismissed among those neutered between 7 and 11 months compared to dogs neutered at younger and older ages was seen among females and males when analyzed separately (Figure 1) and for golden and Labrador retrievers (Figure 2). Using multivariable log-binomial regression, total dismissals and behavioral dismissal were about 40% less likely in dogs neutered between 7 and 11 months than among dogs neutered before 7 months (RR = 0.6, 95% CI: 0.4, 0.8, *p* < 0.01 for both) and about 30% less likely than among dogs neutered after 11 months (RR = 0.7, 95% CI: 0.5, 1.00, *p* = 0.05 for both). Interestingly, when age-at-neuter was in the model, there was also a significant difference between breed groups, with both Labrador and golden retrievers at lower risk for total dismissals (RR = 0.7, 95% CI: 0.5, 0.95, *p* = 0.02 and RR = 0.8, 95% CI: 0.6, 0.9, *p* = 0.01, respectively) and behavioral dismissals (RR = 0.7, 95% CI: 0.5, 1.0, *p* = 0.03 and RR = 0.7, 95% CI: 0.5, 0.9, *p* = 0.005, respectively) than the pooled other breeds. Sex was still not a significant predictor of dismissal nor did it modify the effects of age-at-neuter and was not included in either model.

Health-related dismissals did not differ by sex or breed but were significantly less common for dogs neutered at 7–11 months than for dogs who were neutered before 7 months (Table 1). There was no significant difference between dogs neutered between 7 and 11 months and those neutered after 11 months. This pattern was the same for the subset of health dismissals due to orthopedic problems (Table 1). Based on multivariable log-binomial modeling, dogs neutered before 7 months were more than twice as likely to be dismissed for health reasons than dogs neutered at any older age (RR = 2.4, 95% CI: 1.3, 4.2, *p* = 0.005), with no other significant predictors. Dogs neutered before 7 months were more than twice as likely to be dismissed for orthopedic problems than dogs neutered at any older age (RR = 2.2, 95% CI: 1.1, 4.3, *p* = 0.03). Labrador retrievers were at also higher risk for orthopedic-related dismissal than golden retrievers and all other breeds (RR = 3.3, 95% CI: 1.1, 10.2, *p* = 0.04).

## Discussion

Although pet owners are usually encouraged to spay or neuter their household animals, the effects of gonadectomy and the best time at which to perform this procedure are still being explored. Previous research has encompassed a range of age groups, breeds, and health problems, and the results of those studies have been varied, making definitive generalized conclusions challenging. The American Veterinary Medical Association (AVMA)^1^ supports the concept of pediatric/pre-pubertal gonadectomy in order to reduce the population of unwanted dogs and cats, but the AVMA has also acknowledged that veterinarians must consider each animal's case individually and the best information on spay/neuter available at that time (AVMA). For the service dog community, effects of timing of gonadectomy on health and behavior that can result in the dismissal of an animal from training are particularly relevant. We found that the short-term, behavioral and health effects of age-at-neuter differed. The 7 to 11 month age-at-neuter group had the fewest behavioral dismissals while dogs neutered before 7 months had the most health-related dismissals. This was true for both sexes and our breed groups. Breed was associated with dismissal when adjusted for age-at-neuter.

Successful graduation rates ranged from 7 to 70% when dogs were grouped by different age-at-neuter, sex, and breed combinations. Other studies have also found graduation rates between 30 and 50% with behavioral problems as the major reason for dismissal of service dogs during training (17). Most of our dismissals were due to behavioral problems in all groups. A prospective study of neutering before puberty also found behavior as the leading cause of dismissal [reported in Zink et al. (18)]. For behavior, the age-at-neuter group with the least dismissals was 7 to 11 months of age for both sexes. Associations between behavioral problems and age-at-neuter have varied. A study of dogs adopted from a shelter in NY found that barking in males, noise phobias and sexual behaviors decreased linearly with increasing age-at-neuter while escaping from the home increased with age-at-neuter. Dogs with an age-at-neuter less than 5.5 months of age had less separation anxiety but more aggression toward household members than those neutered at an older age (11). Another study of more than 13,000 dogs also found that dogs neutered before 6 months had more aggression to family members and that aggression toward family and strangers was less in dogs neutered after 12 months (19). Some studies have not found differences in behavior problems between the age-at-neuter groups we studied (18, 20). Our findings support effects of age-at-neuter on the development and training success of service dogs. This likely extends beyond service dogs, since more than 80% of owners, across cultures, report that their dogs have behavioral problems and this is the most common reason for relinquishing dogs (21). Studies that measure behavior longitudinally over time and that look at specific rather than overall behavioral concerns are needed to better understand the dynamics of neutering, behavioral development, and specific problems and potential corrections.

Our finding of a significantly higher rate of dismissal for health problems, mainly orthopedic, among dogs neutered before 7 months is similar to findings in a number of studies. A higher risk for dogs neutered earlier than 6 months, compared to older groups, was reported for hip dysplasia and cranial cruciate ligament tears among golden retrievers (22) and German shepherds (12). Dogs adopted from a shelter in New York who were gonadectomized prior to 5.5 months of age were more likely to demonstrate hip dysplasia when compared with dogs gonadectomized at a later age (11). Golden retrievers neutered before 12 months were also significantly more likely to experience cranial cruciate ligament tears than those neutered after 12 months and early-neutered male golden retrievers were significantly more likely to have hip dysplasia than late-neutered males (23). In contrast, this pattern was less clear for a study of Labrador retrievers (22), and another study found no significant increase in musculoskeletal disorders or hip dysplasia in prepubertal (<24 weeks old) gonadectomized dogs when compared to dogs gonadectomized after 24 weeks of age (20). Overall, the present study adds evidence to others who have shown a greater risk of orthopedic disease in dogs neutered at <7 months of age, despite the short-term follow-up until dismissal during training. Factors in the development of orthopedic disease may include changes in the production of sex hormones and therefore growth in young animals, as well as a possible delay in the closure of growth plates, resulting in orthopedic changes.

Other health problems were noted more frequently in the youngest SFSD age group when compared with the older age-at-neuter groups, although these were a very uncommon reason for dismissal in our study. Most health concerns that have been studied for an association with neutering occur much later in life than during the early training period that we studied (11, 24). It would be ideal to follow these dogs over their lifetimes to gain a more complete picture of health risks. However, dogs are currently not traced after they leave the SFSD program.

On the subject of breeds, our results suggested that Labrador and golden retrievers were less likely than other pooled breeds to be dismissed for behavior problems. This was not surprising, as these two retrievers have commonly and successfully been used as service animals in the past, due in part to their behavior. Within each age-at-neuter group, Labrador retrievers had a greater risk for dismissal for orthopedic disease than golden retrievers or other breeds. This was unexpected, particularly as the Orthopedic Foundation for Animals^2^ reports both hip dysplasia and elbow dysplasia to be more common in golden retrievers than in Labrador retrievers (OFA). Given the absence of substantial genetic history of the dogs in the SFSD study group, it is difficult to determine how much of this finding might be attributed to heritable factors which are well recognized to predispose to a number of orthopedic problems. It is unlikely, though, that breeders would have utilized animals with a history of orthopedic problems for breeding purposes, and other health and environmental factors, such as diet, exercise, and hormones, are important contributors to the development of hip dysplasia (25).

This study had several limitations. Because this study's population was primarily made up of the two retriever breeds, generalization to the dog population as a whole may be challenging. Both breeds are popular as pets, though, making the consequences of neutering relevant to many pet owners and private practice veterinarians. Differences between the necessary qualities of a service dog and qualities of a pet may also affect the generalization of the results here to other, non-service dogs. For example, the owner of a household pet may find a dog's health problem (e.g., overbite) negligible but a behavior problem (e.g., strong prey drive) more challenging.

Dogs did not enter the program at the same age and were not randomly assigned to the time of neutering. Their experiences outside the SFSD program could have affected their behavior or overall health. Any characteristics predictive of training success, that also led to choice of neutering time, could have biased results. For example, puppies demonstrating health or behavior problems prior to their neutering could have been dismissed, leaving more successful dogs in the older age groups. However, most neutering times resulted from overall policies in place at certain times and so this was less likely. Dogs neutered later may not have been checked for hip dysplasia or completed training until later in their lives, providing them a longer period during which to develop health or behavior problems compared to dogs neutered at an earlier age. However, we found the opposite; a greater incidence of orthopedic and health dismissals among younger dogs. The effects of neutering before 7 months may be even larger than our estimates.

Misclassification bias was a concern for the behavior dismissal group, where trainer subjectivity may have affected the decision to classify each dog as having behavioral problems. Additionally, misclassification bias may have been introduced when the authors interpreted the recorded data for each dog, as in when a reason for dismissal had to be determined. The elimination of more than 90 dogs—more than a quarter of the initial study population—from analysis due to missing information may also have affected the results. The data may also have been affected by unmeasured confounders, potentially including genetics, environment, and the type of training during the dog's puppy-raising period.

Future studies could expand upon these findings by randomly assigned age-at-neuter and using standardized methods to measure behavioral problems. Also, if study population sizes were larger, it would also be helpful to subdivide the behavior group so that specific behaviors (i.e., fear, aggression, unwillingness to perform tasks) might be analyzed separately. Similar studies considering multiple cohorts of service dog candidates at different training organizations would also be valuable in drawing conclusions applicable to a wider range of animals, potentially including more breeds, under a variety of different living and training conditions.

## Conclusion

This study suggests that there is a relationship between dogs' age-at-neuter and their subsequent behavior and health. Neutering between 7 and 11 months of age may represent a desirable window for service dogs in training. Orthopedic problems may be more pronounced in dogs neutered at less than 7 months of age, and this may be particularly true of Labrador retrievers.

## Data Availability Statement

The datasets generated for this study are available on request to the corresponding author.

## Author Contributions

MZ collected data, performed data analysis, and composed parts of the manuscript. VC initiated the study along with MA and helped with data analysis and manuscript editing. MA and EG collected data. LH performed data analysis, composed parts of the manuscript, and helped with study design and manuscript editing.

### Conflict of Interest

The authors declare that the research was conducted in the absence of any commercial or financial relationships that could be construed as a potential conflict of interest.

## Footnotes

^1^American Veterinary Medical Association website, *Pediatric Spay/Neuter of Dogs and Cats*. Available online at: https://www.avma.org/KB/Policies/Pages/Pediatric-Spay-Neuter-Dogs-And-Cats.aspx (accessed August 3, 2019).

^2^Orthopedic Foundation for Animals website. Available online https://www.ofa.org/diseases/breed-statistics#detail (accessed July 28, 2019).
